# Single Neurons in M1 and Premotor Cortex Directly Reflect Behavioral Interference

**DOI:** 10.1371/journal.pone.0032986

**Published:** 2012-03-12

**Authors:** Neta Zach, Dorrit Inbar, Yael Grinvald, Eilon Vaadia

**Affiliations:** 1 Biological Basis of Behavior Program, School of Arts and Sciences, University of Pennsylvania, Philadelphia, Pennsylvania, United States of America; 2 Interdisciplinary Center for Neural Computation, Faculty of Exact Science, Hebrew University, Jerusalem, Israel; 3 Physiology Department, Faculty of Medicine, Hebrew University, Jerusalem, Israel; 4 Edmond and Lily Safra Center for Brain Sciences, Department of Medical Neurobiology, Institute for Medical Research Israel-Canada, Faculty of Medicine, Interdisciplinary Center for Neural Computation, Hebrew University, Jerusalem, Israel; Stanford University, United States of America

## Abstract

Some motor tasks, if learned together, interfere with each other's consolidation and subsequent retention, whereas other tasks do not. Interfering tasks are said to employ the same internal model whereas noninterfering tasks use different models. The division of function among internal models, as well as their possible neural substrates, are not well understood. To investigate these questions, we compared responses of single cells in the primary motor cortex and premotor cortex of primates to interfering and noninterfering tasks. The interfering tasks were visuomotor rotation followed by opposing visuomotor rotation. The noninterfering tasks were visuomotor rotation followed by an arbitrary association task. Learning two noninterfering tasks led to the simultaneous formation of neural activity typical of both tasks, at the level of single neurons. In contrast, and in accordance with behavioral results, after learning two interfering tasks, only the second task was successfully reflected in motor cortical single cell activity. These results support the hypothesis that the representational capacity of motor cortical cells is the basis of behavioral interference and division between internal models.

## Introduction

Learning a new task or information includes an acquisition phase of direct interaction with the learned object, followed by an ‘offline’ consolidation into long term memory storage [1]. This process was also shown specifically for sensorimotor tasks, including ∼6 hour long consolidation [2], [3], [4], and LTP in the primary motor cortex (M1) [5], [6]. Indeed, post-acquisition task responses were shown in M1 and in the premotor cortex (together- *the motor cortices;*) [7]–[12].

During the period of consolidation and the formation of a long term memory trace, we sometimes see *behavioral interference*- the disruption of consolidation and subsequent retention by the acquisition of another task. Importantly, not all tasks behaviorally interfere with each other. For example learning a visuomotor rotation task followed by force field adaptation task [13], [14], or a visuomotor rotation task followed by an arbitrary association task [15] did not lead to any behavioral interference, as indicated by retention of both tasks the following day. A rotation task involves a discrepancy between hand movement and the movement of a cursor on the screen, whereas arbitrary association involves movement direction cues starting before movement onset, with hand movement and cursor movement aligned,. In contrast, learning two opposing rotations did lead to behavioral interference, as indicated by lack of retention of the first task [16].

When performing a motor task, the subjects utilize an *internal model*, a computation of the input-output transformation that needs to be completed in order to interact successfully with the world. The fact that some tasks interfere with each other suggests that they utilize the same internal model. The fact that some tasks do not interfere with each other's acquisition suggests that there are several internal models [17]–[20], although the division between them is not yet elucidated. Therefore, studying behavioral interference between tasks offers a glimpse into the functional division of sensorimotor performance.

Currently, the boundaries separating different internal models are unclear and neither are their neural correlates. In this study we attempted to look for the neural basis of behavioral interference between sensorimotor tasks at a single cell level. To do so, we compared the activities of single cells in the motor cortices after learning two interfering tasks (visuomotor rotation and opposing visuomotor rotation) to the activity after learning two noninterfering tasks (visuomotor rotation and arbitrary association), as well as to learning either of the tasks alone.

We found that the after learning noninterfering tasks, activities of cells in M1 and premotor cortices reflected both tasks, whereas after learning two interfering tasks, only the latter task was reflected in neural activity. These findings suggest that single cell activity in motor cortices reflects memory processes, and show a connection between neuronal representation and behavioral interference.

## Results

To look at behavioral interference at the level of single motor cortical cells, we compared neuronal activity before and after learning two novel sensorimotor tasks (rotation and arbitrary association) compared to learning either of these tasks alone. We expected these tasks to be noninterfering and therefore that both be consolidated and recalled successfully the following day. In contrast, we looked at activities before and after learning two visuomotor rotations (rotation for short; rotation followed by rotation of opposing degree) where we expected behavioral interference leading to inferior retention of the first task on the following day.

### Behavior

**Figure 1:**
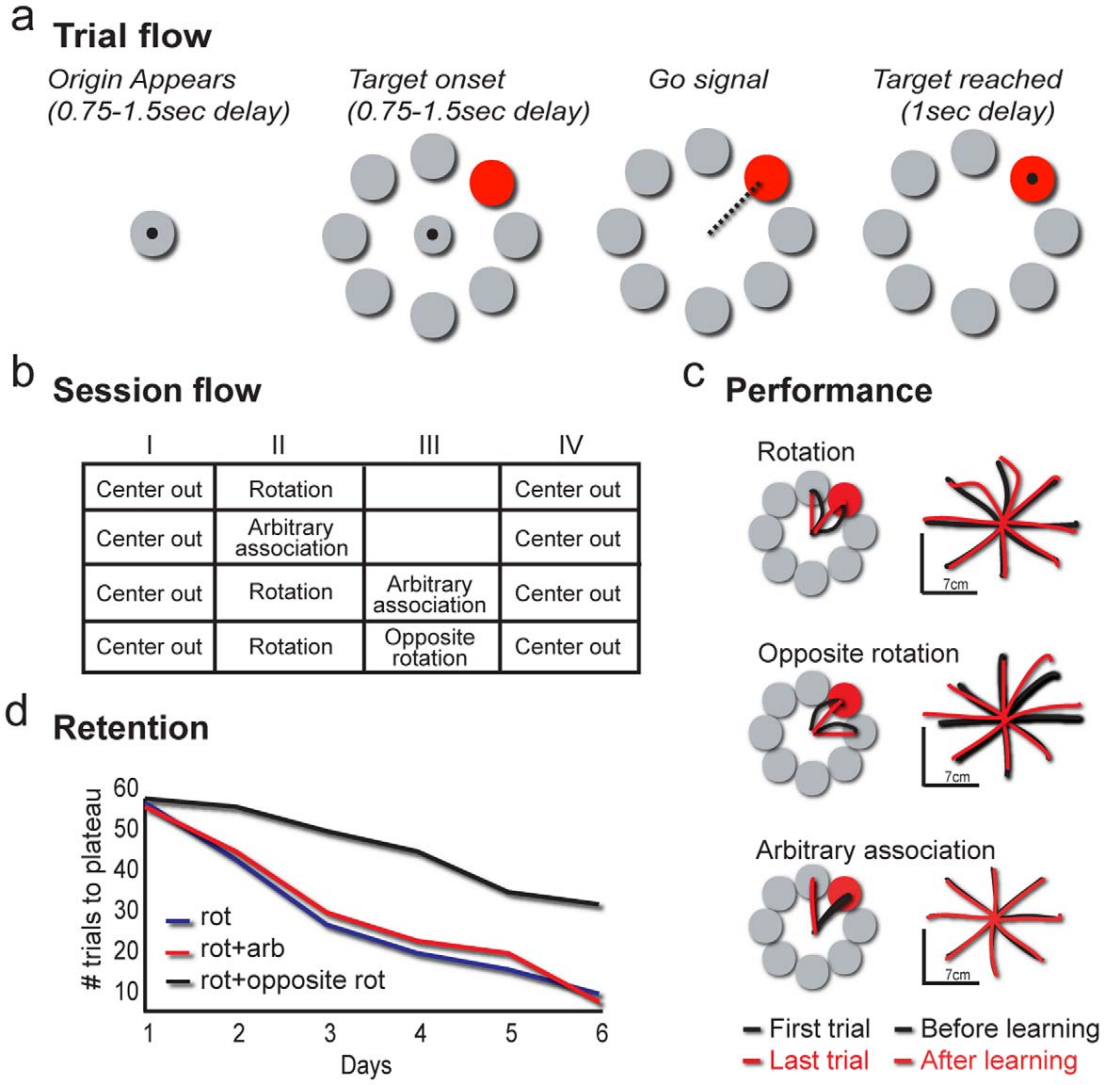
Experimental design and behavioral results. a) Trial flow (left to right): Each trial, in all tasks, always began with the cursor in the central origin. Following a 750–1500 ms delay, 8 targets appeared, 7 white (on a black screen), one colored (target onset; TO; the color was blue, green, red, or magenta). Following a second 750–1500 ms delay the origin disappeared, cueing movement (GO signal). Primates had to reach the target within 1000 ms and hold the curser steady at the target circle for 1000 ms. (b) Session flow. The subjects performed a block of 200 trials of center-out task before and after the novel (learning) task, which was either rotation alone, arbitrary association alone, rotation followed by arbitrary association or rotation followed by the opposing rotation task. Each block comprised 200 trials, each trial according to the trial design in 1a (c) Task performance in the different tasks. Left: example of the first trials performed during learning of rotation (top), rotation followed by opposite rotation (middle; second rotation) or arbitrary association (bottom); each task included only two movement directions. Right: examples of performance during the center-out task before and after learning the novel task. (d) Retention of the rotation task. Measured by the number of trials to reach 100% performance, for monkey M. Black lines represent retention after sessions of rotation alone; red, retention after sessions where rotation was followed by arbitrary association; and blue, retention after session where rotation was followed by opposite rotation. Note that retention after opposite rotation was slower than retention in other cases.

As described in the Methods section, the primates started and ended each session with a standard 8 directions center-out reaching task (Figure 1a), where one of the eight circles is colored (blue, green, red or magenta), cueing the target for movement. After the first block of 200 center-out trials, they were presented with the principal manipulation, comprised of one or two learning blocks (described in Figure 1b): 1. A ***rotation*** task 2. An ***arbitrary association*** task. 3. Rotation followed by arbitrary association. 4. Rotation followed by an ***opposite rotation*** task (*e.g.*, CW vs. CCW). Each acquisition block comprised of 200 trials with two possible movement directions and two possible target colors presented pseudorandomly. After the learning block or blocks, the primates were presented again with another block of the center-out trials.

During the rotation task (performed alone as in manipulation set 1 or before another task as in manipulation sets 3 and 4), target indicated curser movement direction. However, there was a constant 45 degrees discrepancy between the hand movement and the curser movement. Therefore to move the curser, the hand had to move in a 45 degree discrepancy. Target color was either blue or red, but target color was meaningless. As is typical for this task, the hand trajectories were initially curved and then straightened as learning progressed (black lines show the first trials in Figure 1c, left). When the primates were switched back to the center-out task after learning, they demonstrated aftereffects (Figure 1c-right and Figure S1c) in which the hand movements were initially curves due to expectation of a rotation task. As shown previously for both humans and other primates, aftereffects were specific to the movement directions used during the rotation task, and gradually disappeared in less than 5 trials. To avoid any confounding effects, all analyses of neuronal data were done on later trials, when the aftereffects were no longer evidenced. After excluding these initial trials, behavior was indistinguishable from the performance of the center-out task before learning in terms of directional error, velocity profiles and reaction times (p>0.3, Mann-Whitney U test, for all comparisons, Figure S1d, e). In general, beside aftereffects in the first few trials after learning, none of the novel tasks had an effect on performance of the center-out task after learning, as compared to the first center-out block (p>0.35, Mann-Whitney U test, for all comparisons).

To examine consolidation of the rotation task, and assess the possibility of behavioral retrograde interference between rotation and arbitrary association tasks, we looked at retention on the following day in primates that had been trained in learning rotation followed by arbitrary association. Retention was evaluated by the number of trials required to reach stable performance of at least 20 trials (plateau performance). Figure 1d shows that, similar to our previous study on human subjects [15], when primates learned a rotation task followed by an arbitrary association task, retention of the tasks was similar to that observed after learning only one of the tasks (p = 0.42, χ^2^, for a rotation task alone vs. with an arbitrary association task), indicating no interference between these tasks. In addition, there were no differences in the learning curves (Figure S1a). In contrast, when primates performing two rotation tasks of opposing angular deviations (CW vs. CCW) were evaluated the following day, we found inferior retention of the first rotation task (p = 0.0047, χ^2^; Figure 1d, Figure S1a), indicating that these two rotation tasks showed interference to consolidation of the first task as shown previously for humans [14], [15].

To summarize, monkeys and human employ similar strategies for motor learning, showing behavioral interference when learning two opposing rotations, but not when learning rotation followed by arbitrary association.

### Multiple task representations after learning rotation and arbitrary association

In order to elucidate the motor cortices' response after learning two non-interfering tasks, we compared the activity of single neurons in M1 and premotor cortex (termed *motor cortices*), before and after performing rotation alone, arbitrary association alone, or rotation followed by arbitrary association. Comparison was made for each neuron, during the well trained blocks of the center-out task, during two epochs of each trial: (1) from 0–750 ms after *Target Onset* (*TO*) and (2) from 250 ms before *Movement Onset (MO)* to 500 ms after movement onset. Within each epoch, activity was examined separately for 200 ms time bins with a 50 ms interval (i.e., 0–200, 50–250,100–300…).

**Figure 2:**
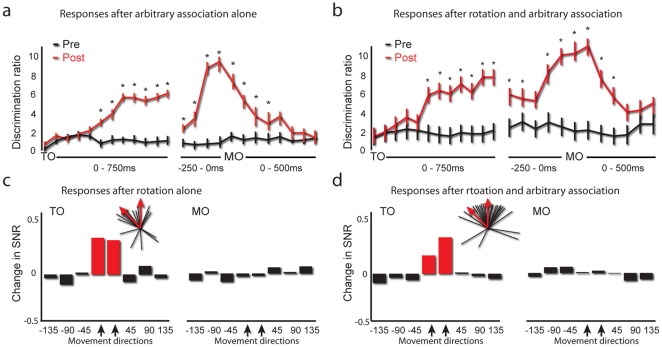
Simultaneous representation of multiple tasks after learning rotation and arbitrary association. (a, c) An increase in discrimination ratio for target colors (i.e. color sensitivity) after learning an arbitrary association task, either alone (n = 140 cells; part a) or after learning both rotation and arbitrary association tasks (n = 317 cells; part c). Black lines represent activity before learning, red lines after learning. Error bars indicate ±1 SEM. Stars indicate a significant discrimination ratio. (b,d) Changes in the SNR for different movement directions for a rotation task either alone (n = 194 cells; part b) or followed by an arbitrary association task (n = 317; part d). SNR changes were normalized to be between −1 to 1 (0<SNR indicates higher SNR after learning). Red bars indicate directions in which SNR significantly increased after learning. Directions are aligned according to distance from the directions used during the novel task. Inset, black lines represent the distribution of preferred directions (PDs) of neurons that significantly increased their SNR. Red arrows mark directions used during learning.

**Figure 3:**
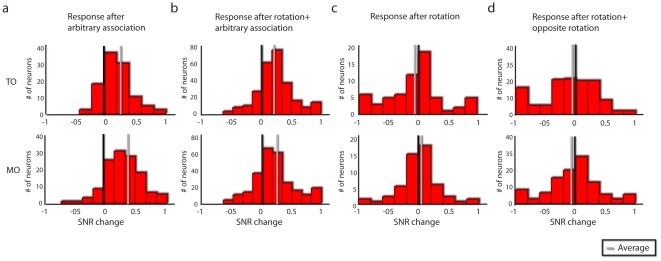
Additional analysis of color sensitivity. Change in the SNR for different target colors during the TO epoch (upper panel) and MO (lower panel), where positive values indicate an increase in SNR after learning: (a) an arbitrary association task alone; (b) arbitrary association following rotation; (c) rotation alone or (d) rotation followed by the opposite rotation. Grey lines indicate the mean of the post-learning distribution, black lines represent zero change. Note that learning an arbitrary association, but not a rotation, resulted in increased SNR.

Figure 2 and 3 shows the neuronal activity of the population of cells, before and after learning rotation followed by arbitrary association, as compared to learning only one of these tasks. As we previously reported [12], learning an arbitrary association task alone leads to an emergence of sensitivity to the colors that were used in the arbitrary association task. This occurs despite the fact that during center-out trials, when the measurements were made, color was irrelevant. We investigated the appearance of such color sensitivity after learning either arbitrary association alone or after learning both rotation and arbitrary association task. To do so, we calculated the number of neurons with differential responses to the different target colors, as measured by ANOVA, divided by the number expected by chance (*Discrimination ratio*, see Methods).

**Figure 4:**
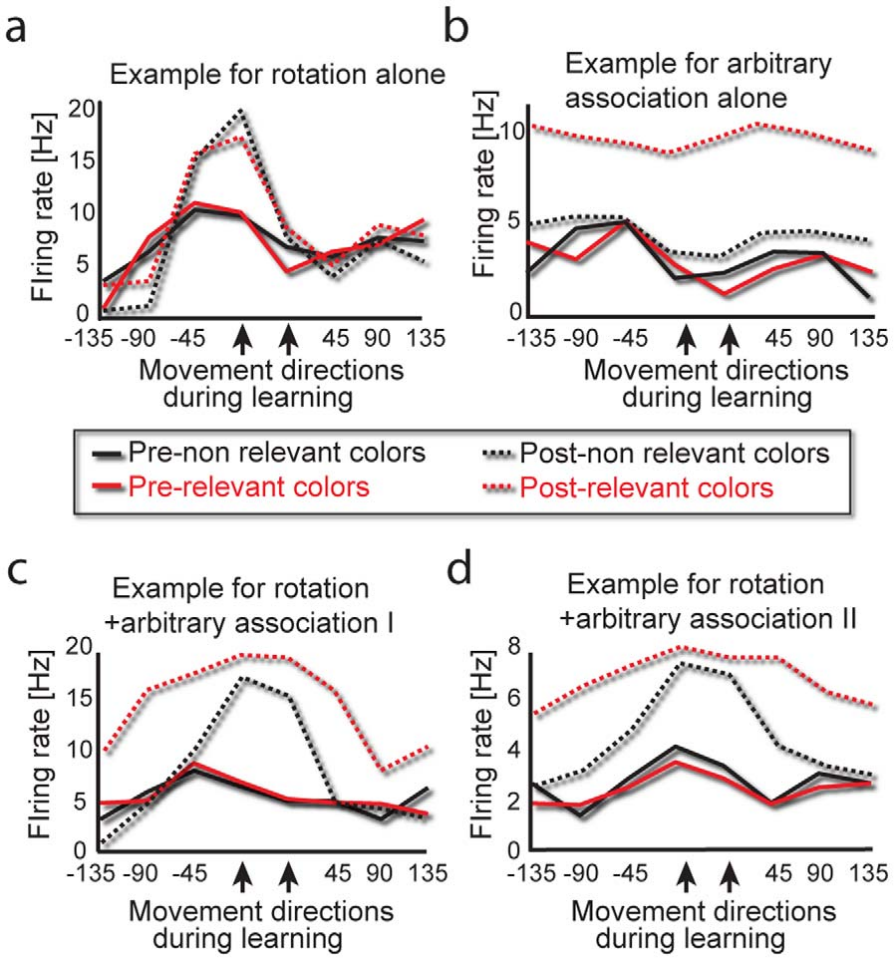
Examples of simultaneous representation in single neurons. Examples of single neuronal responses during the center-out task before and after learning of (a) rotation alone, (b) arbitrary association alone or (c–d) rotation followed by arbitrary association. Red indicates center-out responses to target colors used during the learning blocks, black indicates responses to target colors not used. Solid lines represent activity before learning; dashed lines represent activity after learning. Directions are aligned according to distance from the direction used during the learning blocks.

As shown in Figure 2a, for the sessions in which only arbitrary association was learned (n = 140 neurons recorded), there was an increase in the discrimination ratio of up to six fold during TO and ten fold during MO. This increase was significant from 250–750 ms after TO and −250 ms to 100 ms around MO. The percentage of neurons with discriminative responses to the different colors during each of the time bins is specified in the figure. Up to 18% of the neurons during TO and up to 20% around MO showed significant color discrimination after learning. An example of a single neuron showing a higher post-learning firing rate to the target colors that were used during learning is shown in Figure 4b.

The same color sensitivity also emerged after learning the rotation task followed by the arbitrary association task (n = 317 neurons; Figure 2b); the discrimination ratio increased up to 7.5-fold after TO and up to 11-fold during MO. This increase was significant for 250–750 ms after TO and −100 ms to +150 ms around MO. Up to 21% of the neurons after TO and up to 21% around MO showed significant color discrimination after learning. This increase did not differ from learning the arbitrary association alone (p>0.3, χ^2^). These results suggest that after learning rotation followed by arbitrary association, response to the arbitrary association task was evidenced.

In order to verify the results associated with learning the arbitrary association task, we also looked at the signal-to-noise ratio (SNR; see Methods) for the target colors used during the arbitrary association task vs. the colors not used (only used during the center-out task, where they were irrelevant to task performance), before and after learning (during the time bins 350–550 ms after TO and −150 ms to 50 ms around MO). For the sessions in which an arbitrary association task was learned, either alone (Figure 3a) or following a rotation task (Figure 3b), there was a significant increase in the post learning SNR for the colors used during arbitrary association, compared to before learning and to the color not used in the arbitrary association task (p<0.01, Kolmogorov-Smirnoff test; corrected for multiple comparisons Figure; 3a–b).

As a control, we assessed color sensitivity after learning either a rotation task or two opposing rotation tasks, where the target colors were identical to the arbitrary association task, but were irrelevant to the task. No differences in the SNR before or after learning were found for the target colors, for either rotation alone or two opposing rotations (p>0.35 for both, Figure 3c–d). Also, there was no increase in color discrimination after learning one rotation or two opposing rotations (p>0.5; not shown). This finding suggests that the response to the relevant target colors emerges only after learning the arbitrary association, in which color is the relevant parameter.

Next, we examined the neuronal changes accompanying learning of a rotation task. A previous study showed a post-learning increase in SNR specific to the movement directions that were perturbed during the rotation task [7]. Therefore, we looked at SNR changes for different movement directions before and after learning a rotation task, either alone or preceding an arbitrary association task. Replicating these results, we found that learning rotation alone led to a very specific change in neuronal representation (n = 197 neurons; Figure 2c): 1) specific to the directions used during the rotation task (p = 0.006, χ^2^, for learned directions, p>0.3 for all other directions); 2) specific almost exclusively to neurons whose preferred directions (PD) were close (±22.5°) to these directions (the PDs of neurons with increased SNRs were non-homogeneously distributed, p = 0.002 by Rao's spacing test, n = 17 neurons; see Figure 2c, inset) and 3) specific only to the TO epoch, before movement was initiated (p>0.3 for all SNR changes during MO). An example of a single neuron showing a higher post-learning firing rate to the directions used during learning is shown in Figure 4a.

The same neuronal representation emerged after learning rotation followed by arbitrary association. As shown in Figure 3d, we found the very same effect on neuronal activity. An SNR increase, specific: 1) for directions used during the rotation task (p = 0.007 for learned directions, p>0.35 for all other directions); and 2) for neurons whose PD was close to these directions (p = 0.0007 by Rao's spacing test, n = 25 neurons; see Figure 2d, inset and 3) for changes during the TO epoch (p>0.3 for all SNR changes during the MO epoch).

These results indicate that the enhancement in directional representation found after rotation was not impaired by subsequent learning of an arbitrary association task.

In order to further verify the enhancement of directional representation found after learning the rotation task (Figure 2c,d), we measured the SNR for different movement directions before and after learning the arbitrary association task alone, where movements were made to the same direction, but without any perturbation. As shown in Figure S2, no SNR increase was observed for any of the directions or epochs (p>0.3 for all directions), and only a few neurons (n = 4 neurons during the TO epoch) showed an increase in the SNR (with no trend toward any direction, p>0.3, Rao's test for homogeneous distribution). This finding suggests that an enhanced representation of movement directions manipulated during the rotation task emerges specifically after learning rotation, where direction is the relevant parameter.

The results presented thus far indicate that after learning two non-interfering tasks — a rotation task and an arbitrary association task – the neuronal population of the motor cortex represented both tasks successfully. Specifically, the effects on neuronal activity caused by adaptation to the rotation tasks (Figure 2c) were unaffected by the subsequent learning of the arbitrary association task (Figure 2d). Similarly, the effects on motor cortex representations caused by learning the arbitrary association task (Figure 2a and 3a) were unaffected by prior learning of the rotation task (Figures 2b and 3b). To summarize, these results show that after learning two none interfering tasks, the motor cortices are simultaneously representing both tasks.

### Single neurons can represent two tasks simultaneously

Could these multiple representations be achieved not only by the neuronal population as a whole but also by single motor cortical cells? Figure 4 shows two examples suggesting that this is the case. Red lines mark the responses when the target color is one of two colors that were used during the arbitrary association task. Black marks the responses when the target color was one of the two colors that were not used during the arbitrary association task (only used during the center out task).

Neurons responding to the rotation task (Figure 4a) did so by an increase in FR specifically during the TO epoch, specifically for the directions used during the rotation task (i.e. in their PD; marked by an arrow in Figure 4). This firing-rate elevation was found across different target colors.

Neurons responding to the arbitrary association task (Figure 4b) did so by a preferred response- an increase in firing rates- specifically to the colors that were used during the arbitrary association task, regardless of the movement directions. These types of changes were observed for both TO and MO epochs.

Examples of neurons responding to learning both rotation and arbitrary association tasks are shown in Figure 4c, d for the TO epoch. These neurons show the response associated with learning rotation- an increase in FR for the directions used during the rotation task, almost regardless of target color. Also, these two neurons show the response associated with learning the arbitrary association- an increase in FR for the colors used during the arbitrary association task, almost regardless of movement direction. Therefore, these neurons were representing both task parameters simultaneously.

**Figure 5:**
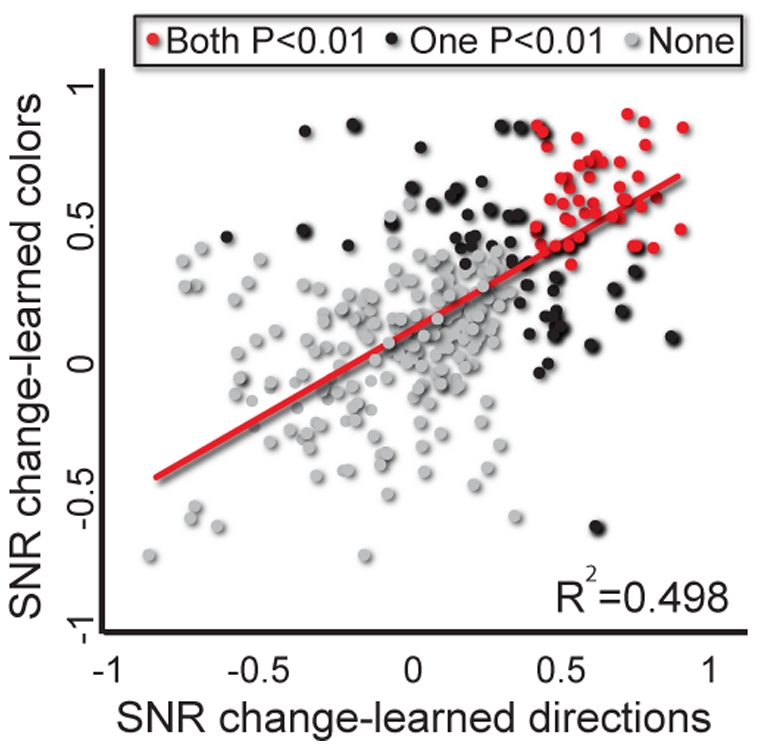
Simultaneous representation by single neurons after learning rotation and arbitrary association. Correlations between representation of the arbitrary association task (SNR change for target colors used during arbitrary association task) and the rotation task (SNR change for directions used during rotation task), by single neurons. Grey dots represent neurons that did not show significant changes, black dots represent neurons that showed significant SNR increases for only one of the tasks and red dots represent neurons whose SNR changed significantly for both tasks.

For the neurons recorded during sessions of both rotation and arbitrary association task, we quantified the number of neurons showing improved representation of movement direction following the rotation task, improved representation of target color following the arbitrary association task, or both (Figure 5). A representation of the rotation task was defined as a significant increase in SNR for the directions used during the rotation task vs. the directions not used. A representation of the arbitrary association task was defined as a significant increase in SNR for the target colors used during the arbitrary association task vs. the colors not used.

We found that during the TO epoch, 96 out of 317 neurons significantly represented at least one of the tasks, out of which 68 (21.4%) represented rotation (movement direction) and 74 (23.3%) represented arbitrary association (target color). If the representation of rotation and arbitrary association tasks was independent, then the chances of one neuron representing both tasks are 0.233*0.214 = 0.05 (5% of the population, 16 neurons). However, we found 46 neurons representing both rotation and arbitrary association tasks (red dots; 46/317 = 14.5% p<0.01 more than 5%, χ^2^). In other words, 48% of the 96 neurons representing at least one of the tasks actually represented both of them. Out of these 46 neurons, only 1 had a significant interaction between direction and color discrimination (as measured by a two-way ANOVA, p>0.5, χ^2^), suggesting that the two parameters were separable. Together, these results show that simultaneous representations of both tasks at the single cell level were common and significant.

Furthermore, there was a significant correlation between the magnitude of each neuron's representation of rotation and its representation of arbitrary association (r^2^ = 0.498; p<0.001). This correlation shows that neurons representing one of the task parameters were more likely to also represent the other. Together, these results demonstrate multiple representations of tasks in a substantial fraction of motor cortical neurons.

Within the motor cortices, neurons representing both tasks were found in both M1 and premotor areas of the two primates. Figure S3 shows the surface map of recording sites (for Monkey M; gray dots- arbitrary association alone, black- rotation alone, red- both). Neurons of all types were widely distributed, extending from the central sulcus to the arcuate sulcus and including both ventral and dorsal premotor areas. This was also true for Monkey K (n = 10, 7, 5 neurons representing both rotation and arbitrary association, only arbitrary association or only rotation, respectively, with all types distributed across M1 and premotor cortex). These findings indicate that both M1 and premotor neurons respond to learning by representing both tasks simultaneously.

To conclude, after learning rotation and arbitrary association, single neurons in the motor cortices represented multiple tasks simultaneously.

### No simultaneous representation after learning rotation and opposite rotation

We then turned to compare the responses after learning two tasks that interfere with each other's consolidation; namely, two opposing rotation tasks (CW vs. CCW, see Figure 1d). We examined firing rates during the performance of the center-out task after learning a rotation task followed by the opposite rotation.

**Figure 6:**
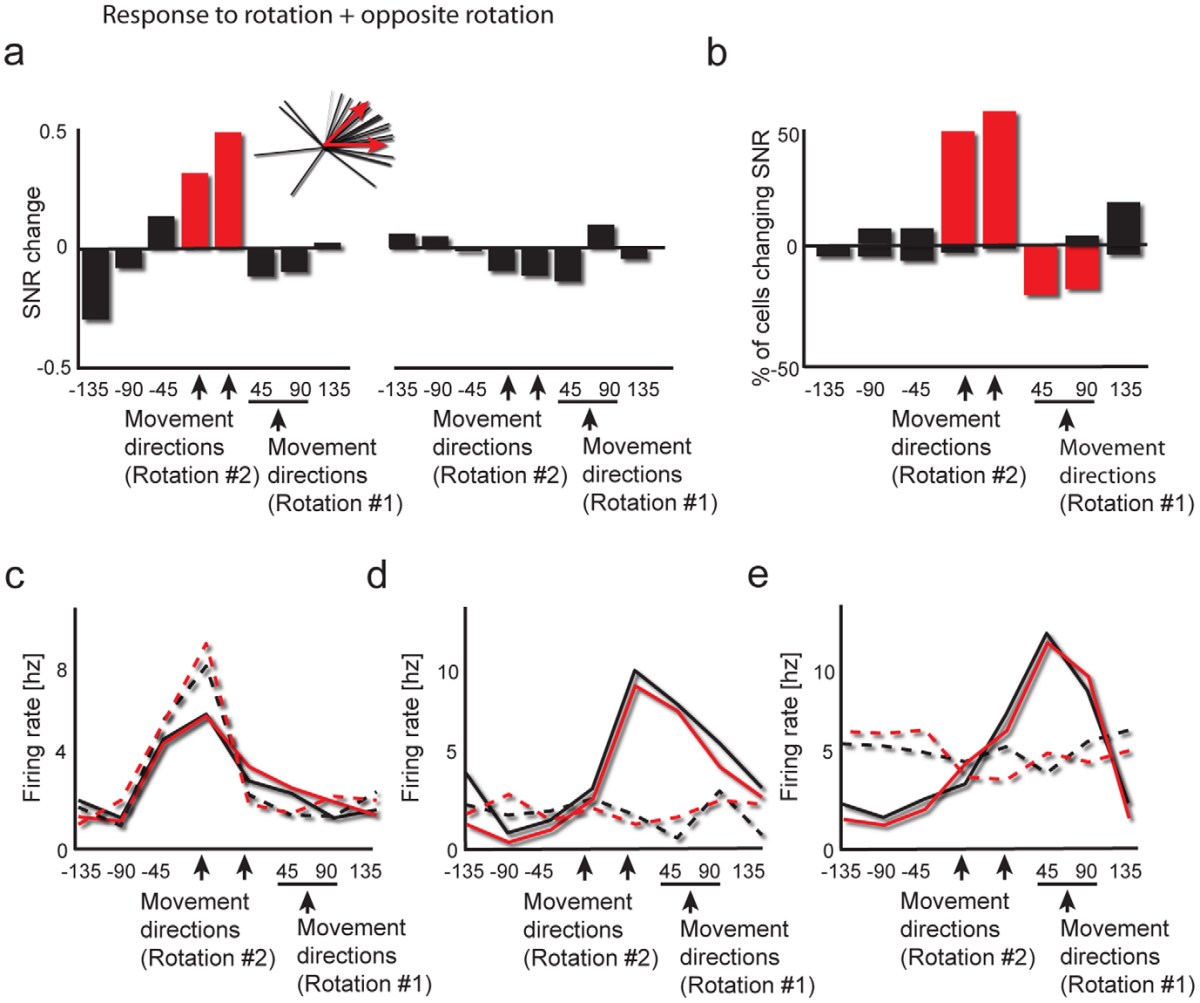
The lack of simultaneous representation after learning rotation followed by opposing rotation. (a) SNR changes for rotation followed by opposite rotation tasks (n = 140 cells). Notation same as Figure 2c. Note that SNR elevation is restricted to the directions used during the second rotation. (b) Percentage of neurons significantly increasing or decreasing their SNR after learning (during the TO epoch). Red marks directions where a significant portion of the neurons changed their SNR. (c–e) Examples of single neurons, notation same as Figure 4. (c) Neuron with a pre-learning PD that was close to the directions used during the second rotation. (d–e) Two neurons whose pre-learning PDs were close to the directions used during the first rotations.

Figure 6a shows the emergence of a representation of the second task after learning. During the TO epoch there was an increase in the SNR for the movement directions that were used during the second rotation (p = 0.003, χ^2^, for directions used during the second rotation, Figure 6a), and neurons whose PDs were close to these directions changed their SNR (p = 0.007 by Rao's spacing test, n = 17 neurons, see Figure 6a–b). An example of such a neuron is shown in Figure 6c, with firing-rate increases similar to those observed after learning only one rotation task (Figure 4a).

In contrast, responses associated with the first rotation were not present after learning; there was no increase in SNR for the directions used during the first rotation (p>0.5). In fact, ∼20% of the neurons whose PD was close to these directions showed a significant decrease in their SNR during TO (p<0.05, χ^2^, compared to other directions, Figure 6b). This effect was specific for neurons whose PD was close to that used in the first rotation; the general direction representation was unaffected (Figure S4). Examples of such neurons are shown in Figure 6d–e. When the second opposite rotation was also learned, instead of firing rate increases, there was a decrease, and many of the cells lost their cosine-like tuning curves.

To summarize, we found several differences between learning two rotation tasks compared to learning a rotation task followed by an arbitrary association task. First, learning a second rotation task, but not an arbitrary association task, led to reduction in retention of the first task the next day (Fig. 1d). These results are supported by previous literature (Zach et al 2005) and suggest that differing rotation tasks compete over common resources, whereas rotation and arbitrary association tasks do not. Looking at these “common resources” we looked at neural representation changes during the center out task after learning. We found that in the case of learning rotation followed by arbitrary association, both tasks were represented after learning at the population level (Fig. 2 and 3) as well as the single cell level (Fig. 4 and 5). In contrast, after learning two rotation tasks, only the latter task was apparent in neural activity after learning (Fig. 6).

Taken together, these results show that two tasks that can be remembered together are also represented together, whereas two tasks that interfere with each other's consolidation are not represented simultaneously by single motor cortical neurons. The results also suggest a connection between single cell representation and consolidation (and retention the next day).

## Discussion

This study shows that when primates learned tasks that did not interfere with each other's consolidation—a rotation task and an arbitrary association task—simultaneous representations of both tasks were apparent in neurons after learning at both the population level and the single-cell level. These simultaneous representations were as common as those of a single parameter or task. In contrast, when learning two interfering tasks - a rotation task and an opposing rotation task - these tasks were never simultaneously represented by the same cortical cells. These findings demonstrate the relationship between brain and mind – between motor cortical representation and behavioral interference with consolidation.

Our findings show that the acquisition of two tasks that can be learned and consolidated together successfully (as evidenced by improved retention on the following day) led to the simultaneous representation of both tasks in neurons. In contrast, two rotation tasks that cannot be learned and consolidated together, were not simultaneously represented in neurons.

When looking at the neural activity associated with the two pairs of tasks, one difference seems of particular importance: rotation and arbitrary association task can be represented by a single motor cortical neuron, whereas an opposing degree of rotation cannot. Rotation and arbitrary association tasks use different, independent parameters - color vs movement direction - and our data show that indeed cells have the representational capacity to separate the parameters and discriminate both successfully.

In contrast, rotation and opposite rotation are represented by two distinct populations of cells, each of which has a preferred direction close to the direction of the rotation- and by contrasting mechanisms- elevation of firing specifically to these movement direction without any change for any other movement directions. Assuming cosine-like directional tuning, each motor cortical neuron cannot hold more than one preferred direction and therefore cannot represent both tasks.

Furthermore, it appears that during the time of consolidation, the motor cortices cannot represent the two opposing rotations by utilizing two separate populations of cells. This may be explained by considering the accumulating evidence that the motor cortices generate their final output as a population. One simple and well supported mechanism for a population output is vectorial summation of cells' firing rate according to their PD [21]–[25]. In population coding by vectorial summation, activity representing two conflicting signals will be averaged into an intermediate message, which will be erroneous. To prevent this from occurring, the second representation will cause the first one, which is no longer relevant, to be eliminated [26]. Subsequently, during learning it is important not only that the cells with the relevant PD will increase their firing rate, but also that there will be no increase for non-relevant directions. Therefore, two opposing rotations cannot be reflected simultaneously by the activity of motor cortical cells. Instead, in order to successfully represent the second task, the activation associated with the first task must be eliminated and indeed it is eliminated- neurally and behaviorally.

This leads us to the following speculative interpretation of our results: that the limitation of representation by the motor cortex is the *cause* for the behavioral difference between the pair of task. That is, that **behavioral interference between tasks stems from the inability of the motor cortices to represent both tasks simultaneously during consolidation**.

Since the appearance of behavioral interference is an indication of the utilization of a common resource- an internal model of the input-output relation between the command and its execution- these results suggest that this common resource is the activity of the motor cortices- Two internal models can co-exist if they can be represented simultaneously.

The theory is also supported by recent findings according to which dual task interference stemmed from involvement of overlapping parts of the frontal and parietal cortices [27].

This theory also explains previous findings; It can explain previous reports of lack of interference between tasks. According to our theory, tasks that can be simultaneously represented will not show behavioral interference. This fits well with finding of lack of interference between rotation and force field adaptation [17]–[30]- the representation of direction and force by motor cortical cells is separable, with direction being represented by the tuning curve width and peak, whereas force is represented by the amplitude [31], [32]. On the behavioral level, we see a separation of kinematic and dynamic aspects of movement; on the neural level, we see separation in neural representation.

By the same token, it can also explain why separate mechanical loads that lead to separate motor cortical responses are indeed utilizing two separate internal models [33].

However, further investigation is needed to formalize this theory. For instance, what is the relationship between the premotor cortex and the primary motor cortex? In this study we found similar responses in both, but that is not sufficient to indicate that they both serve in the same role. Another interesting issue is that of inter-limb transfer and connection between the activity in the two hemispheres.

To summarize, the results presented in this study lead us to suggest that the underlying principle behind both behavioral interference with consolidation of sensorimotor tasks and the division between different internal models, is the inability of the motor cortical neural cell population to represent both tasks simultaneously during consolidation. Therefore, the ability of some tasks to be learned together results from their ability to be reflected together in the motor cortical activity.

## Methods

### Ethics

Animal care and surgical procedures complied with the US National Institute of Health (NIH) Guide for the Care and Use of Laboratory Animals. The study was approved by the Institutional Committee for Animal Care and Use at the Hebrew University, permit number MD-78-03-3.

Details of animal welfare and steps taken to ameliorate suffering were in accordance with the recommendations of the Weatherall report, “The use of non-human primates in research”.

Animals were kept in common yards with enrichment devices. For reinforcement learning reasoning, they were kept under food restrictions during the week. Drops of juice (usually Gerber enriched with baby formula) were provided as a reward for task success. Monkeys enjoyed weekends of full feeding and at all times were not deprived of water. A veterinarian inspected them weakly and performed routine tests. All procedures were sterile and under anaesthesia, with pain relievers.

Two female monkeys *(Macaca fascicularis*, ∼3 kg) were trained on an 8-direction center-out reaching task, using a 2-joint manipulandum at their elbow level. All aspects of animal care were according to Hebrew University Animal Care and Use Committee standards and approved by it. Extracellular recording sessions started after training on center-out task. Signals from 32 moveable electrodes were sorted and sampled at 25 kHz (Alpha-Omega, Nazareth, Israel). Locations of penetrations are indicated in Figure S3.

### Spike sorting

When recording extracellularly, signals coming from different neurons need to be carefully separated to verify that they arrive from one source neuron rather than several. We took several measures to achieve that: 1) Before we commenced recording, we identified (via threshold) and tracked interspike interval histograms for at least 10 minutes before deciding to include them in the recording [34]. 2) The signals coming from different neurons were then separated using Principal Component Analysis according to the shape of the spike wave form [34]. 3) We only included one signal from each electrode and electrodes were spaces at least 0.4 mm apart. 4) waveform had to remain consistent across the entire session or the putative cells were discarded.

### Trial flow

Each trial began with the appearance of an “origin” (Figure 1a, left). The monkey had to use the manipulandum to position a cursor (black dot, Figure 1a) within this circle (0.7 cm radius), for 750–1500 ms (varied randomly). Then, at Target Onset (TO) eight circles (0.7 cm radius) appeared, spaced evenly, as illustrated in Figure 1a. All circles were white except one. During the center-out task, the colored circle (red, green, blue or magenta, selected pseudorandomly) served as the target. After a second delay of 750–1500 ms, the circle at the origin disappeared, cueing the primates to move (‘GO signal’). The primates were rewarded if they moved in a straight trajectory (limited by an invisible corridor, 1.4 cm in width), reached the target within 1.0 s, and held the cursor within the circle for an additional 1.0 s.

### Session flow

During recording, each session started and ended with a block of the center-out task (8 targets, 4 colors, 224 trials for each block), with one or two learning blocks in between, as noted in the middle two columns of Figure 1b. There was no cue to indicate block switching. Learning blocks were either ***arbitrary association*** only, ***rotation*** only, both ***rotation and arbitrary association***, or both ***rotation and opposite rotation*** (Figure 1b), as described below:

### Rotation task (Figure 1c top and middle)

A discrepancy of 45°, clockwise from the target location (CW) in some sessions and counter clockwise (CCW) in others, between hand movement and cursor movement was introduced for two target locations (100 trials for each location, two possible target colors, appearing pseudorandomly). Thus, in order to move the cursor to the target, movements had to be made 45° from the target. Performance was similar for CW and CCW rotations (made for the same target locations but different movement directions). Sessions including *rotation and opposite rotation* were either CW followed by CCW or vice versa. There were no differences in performance that depended on the order of presentation and therefore the sessions were considered together in the analysis.

### Arbitrary association task (Figure 1c, bottom)

The color of the circle, and not its location, indicated the target. For example, a red circle always instructed a movement to the target at 90° (up in Figure 1a, c), regardless of where it appeared. The colored circle could appear in one of two locations and there were two color-direction associations (100 trials for each, pseudorandomized). The primates had to learn to regard color as the relevant parameter and to find the two specific color – direction associations. In sessions including rotation and arbitrary association, the circle's locations and colors and the movement directions were similar for the rotation block and the arbitrary association block. All sessions were repeated daily with the same parameters until one of the novel tasks was fully acquired. Acquisition in each session was assessed by calculating the number of trials required to reach stable performance for at least 20 consecutive trials (Figure 1d).

### Data analysis

The data include *rotation* (n = 127 cells for Monkey K; n = 67 from Monkey M), *arbitrary association* (n = 104 K, n = 36 M), *rotation and arbitrary association* (n = 76 K, n = 241 M) and *rotation and opposite rotation* sessions (n = 40 K, n = 100 M). All cells were recorded in M1 and premotor areas (Figure S3). The average firing rate was higher than 1 Hz and maintained stable firing throughout the entire session. Since there were no differences between subjects in firing rate, variability, or any other neural parameter tested, the neurons of the two primates were analyzed as one group.

The analysis of neuronal activity focused on firing rates as the primates performed the center-out task, once before learning (left column in Figure 1b) and once after learning (right column in Figure 1b). Responses to target color and movement direction were analyzed using ANOVA, signal-to-noise ratio (SNR) and preferred direction (PD) analyses. All analyses were carried out using the same number of trials before and after learning, for 200 ms time bins across TO and MO. In case of rotation and opposite rotation sessions the first 10 trials were excluded to avoid recording responses during *aftereffect*s, as discussed above.

ANOVAs were calculated for each time bin using multi-way ANOVA for color, direction and interaction, before and after learning (for bins of 200 ms). We calculated the discrimination ratio, defined as the number of discriminating neurons (neurons that showed discrimination of either color or direction, without interference, with a significance threshold p<0.01, corrected for multiple comparisons) divided by the number of discriminating neurons expected by chance. Chance level was estimated using bootstrap methods - trials were shuffled and randomly assigned a criterion, and discrimination between these random trials was determined using ANOVA. This process was repeated 500 times, separately for the pre- and post-learning blocks. Significance was determined using the Mann-Whitney U Test (p<0.01, corrected for multiple comparisons).

SNR was calculated by dividing the mean firing rate by its standard deviation. Significance was estimated using χ^2^ (p<0.01, corrected for multiple comparisons). Change in SNR was calculated for each direction and color by:
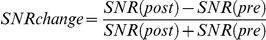
Where *pre* indicates data from the center-out task before learning and *post* data come from the same task after learning. Significance for the population was calculated using a Kolmogorov-Smirnov test, not assuming a normal distribution; (p<0.01, corrected for multiple comparisons). The significance of changes in the percentage of neurons increasing or decreasing SNR was assessed using the bootstrapping method.

PD was calculated by fitting a cosine to the tuning curve of each cell. To ensure the validity of the PDs, only cells with a fit of R^2^>0.55 to a cosine were included in this analysis. The uniformity of the PD distribution was evaluated by a Rayleigh test and homogeneity by Rao's spacing test (significance threshold, p<0.01, corrected for multiple comparisons). Tuning curves and SNR were calculated for the time bins 350–550 ms after TO and ^−^150 to +50 ms relative to MO.

## Supporting Information

Figure S1 **Experimental design and behavioral results.** (a) Learning curves for the rotation task during the different sessions. Blue lines represent performance during rotation sessions, red lines during rotation followed by arbitrary association, black and gray lines, the first and second rotations during the two opposing rotation sessions, respectively. (b) Retention of the rotation task, as measured by directional error of the first rotation trials on each of the learning sessions, for monkey M. Black lines represent retention after sessions of rotation alone; red, retention after sessions in which rotation was followed by arbitrary association; and blue, retention after sessions in which rotation was followed by opposite rotation. Note that retention of rotation that was learned an opposite rotation was slower. (c) *Aftereffects*. Directional errors during peak velocity throughout the first 10 trials of the center-out task (notations as in part a). (d–e) Movement parameters for the center-out task before (black) and after (red) learning. (d) Trajectories (left) and velocity profiles (right). (e) Reaction times. Note that there were no differences in performance before and after learning.
(TIF)

Figure S2 **Directional representation before and after arbitrary association.** SNR change for different movement directions for the arbitrary association task. Notation as Figure 2c–d.
(PDF)

Figure S3 **Surface map for recording locations of cells representing the rotation or arbitrary association tasks (during the TO epoch), taken from monkey M, extracted from MRI analysis (Biospec Bruker, 4.7 T) and verified by skull endocast analysis.** Abbreviations: as, arcuate sulcus; cs, central sulcus; ps, principal sulcus. Black dots: location of cells with increased SNR to directions used during rotation (n = 21). Gray dots: locations of cells with increased SNR to colors used during arbitrary association (n = 20). Red dots: location of cells with increased SNR both for colors used during arbitrary association and directions used during rotation (n = 25).
(TIF)

Figure S4 **Directional representation is unaltered by any of the learning sessions.** (a) Discrimination ratio for the different movement directions before and after learning. Notation as Figure 2a–b. (b) PD distributions before (black) and after (red) learning. Note that none of the learning sessions altered directional representation, in general.
(TIF)
