# Supplementary figures and images for: Single Neurons in M1 and Premotor Cortex Directly Reflect Behavioral Interference
Source: PLoS One. 2012 Mar 12;7(3):e32986. doi: 10.1371/journal.pone.0032986 (PMC3299706; doi:10.1371/journal.pone.0032986)

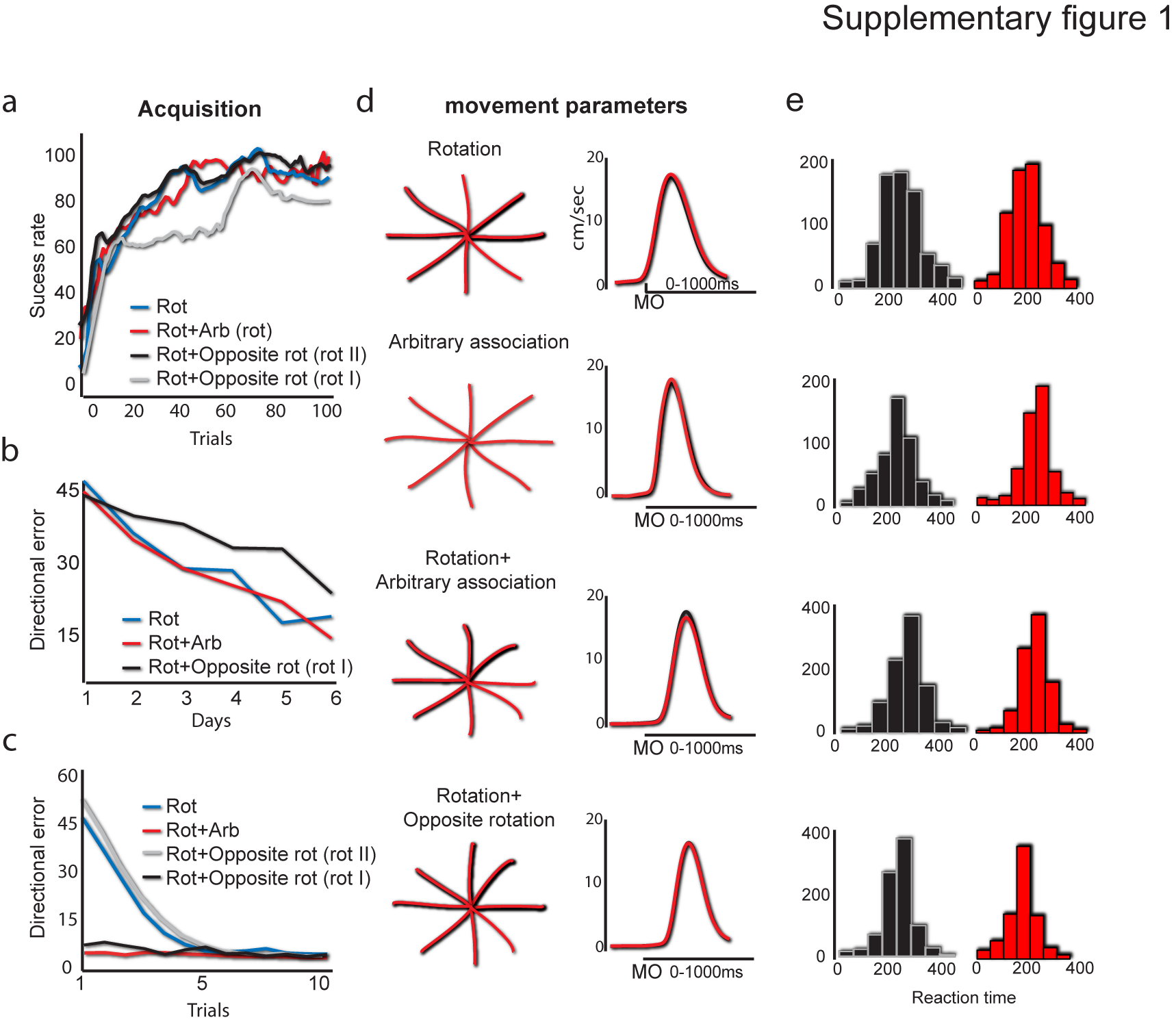

Supplement: Figure S1 — Experimental design and behavioral results. (a) Learning curves for the rotation task during the different sessions. Blue lines represent performance during rotation sessions, red lines during rotation followed by arbitrary association, black and gray lines, the first and second rotations during the two opposing rotation sessions, respectively. (b) Retention of the rotation task, as measured by directional error of the first rotation trials on each of the learning sessions, for monkey M. Black lines represent retention after sessions of rotation alone; red, retention after sessions in which rotation was followed by arbitrary association; and blue, retention after sessions in which rotation was followed by opposite rotation. Note that retention of rotation that was learned an opposite rotation was slower. (c) Aftereffects. Directional errors during peak velocity throughout the first 10 trials of the center-out task (notations as in part a). (d–e) Movement parameters for the center-out task before (black) and after (red) learning. (d) Trajectories (left) and velocity profiles (right). (e) Reaction times. Note that there were no differences in performance before and after learning. (TIF) [file pone.0032986.s001.tif]

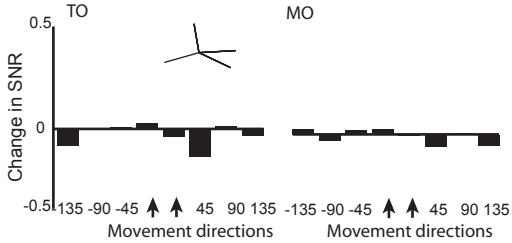

Supplement: Figure S2 — Directional representation before and after arbitrary association. SNR change for different movement directions for the arbitrary association task. Notation as Figure 2c–d. (PDF) [file pone.0032986.s002.pdf]

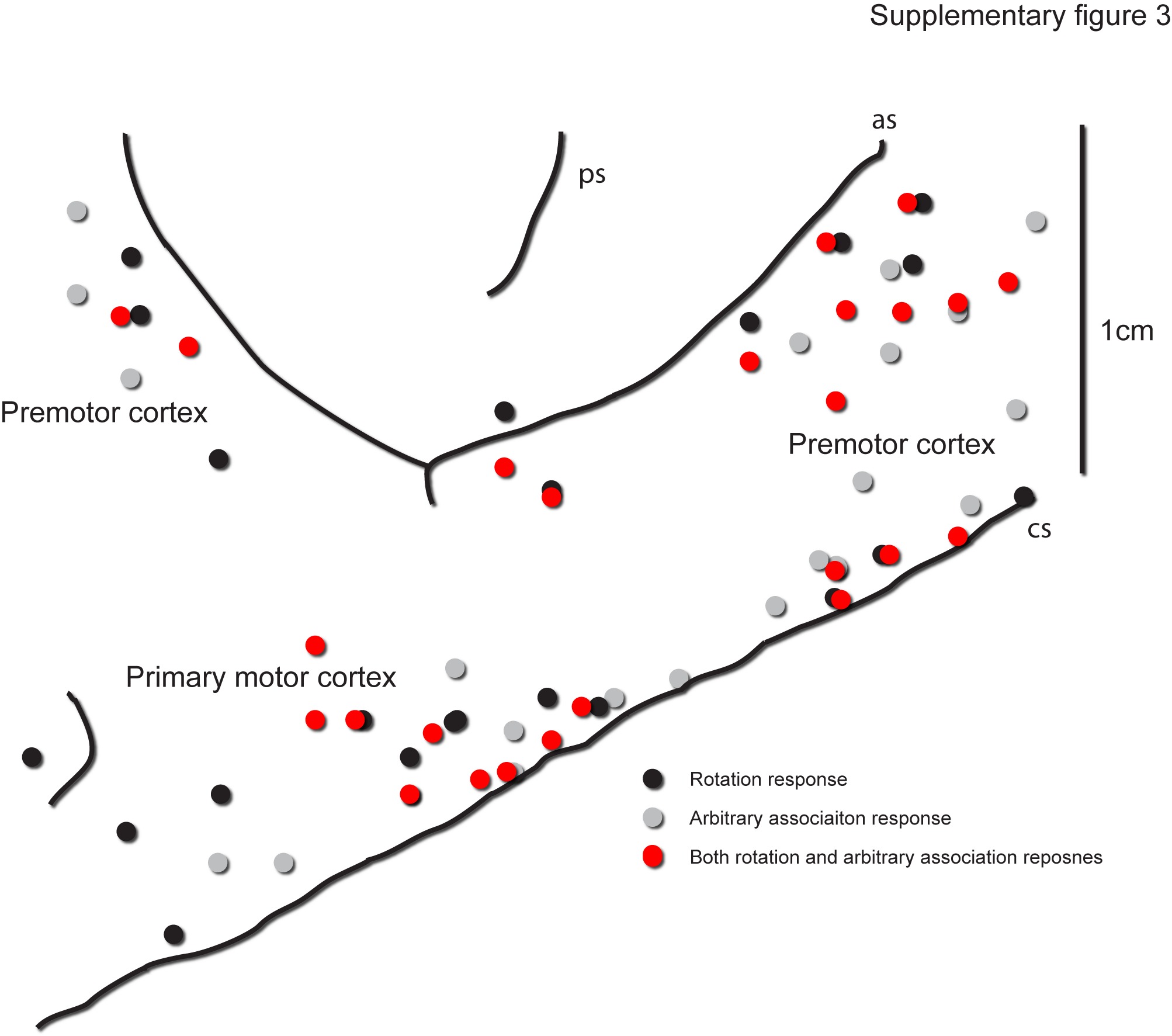

Supplement: Figure S3 — Surface map for recording locations of cells representing the rotation or arbitrary association tasks (during the TO epoch), taken from monkey M, extracted from MRI analysis (Biospec Bruker, 4.7 T) and verified by skull endocast analysis. Abbreviations: as, arcuate sulcus; cs, central sulcus; ps, principal sulcus. Black dots: location of cells with increased SNR to directions used during rotation (n = 21). Gray dots: locations of cells with increased SNR to colors used during arbitrary association (n = 20). Red dots: location of cells with increased SNR both for colors used during arbitrary association and directions used during rotation (n = 25). (TIF) [file pone.0032986.s003.tif]

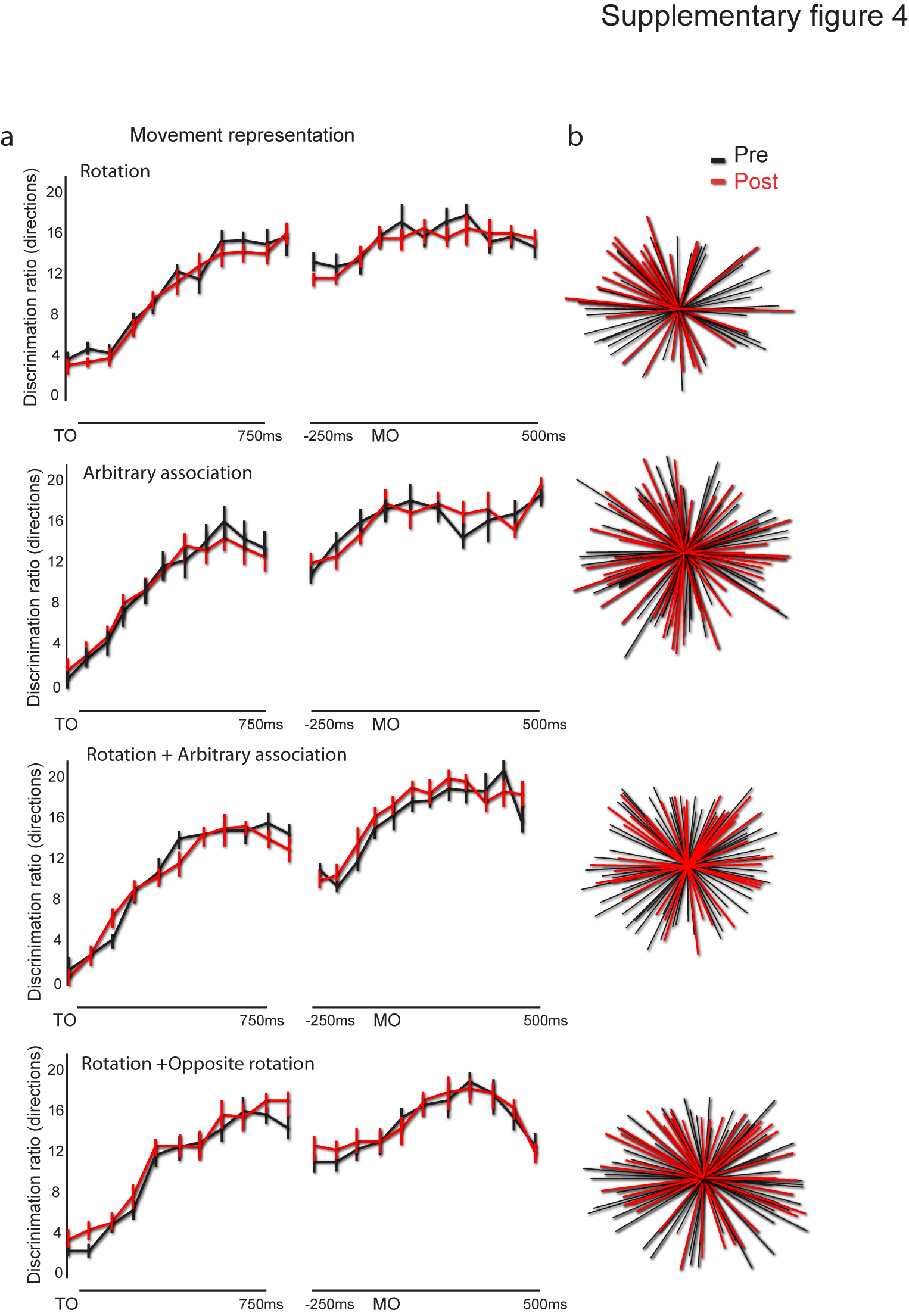

Supplement: Figure S4 — Directional representation is unaltered by any of the learning sessions. (a) Discrimination ratio for the different movement directions before and after learning. Notation as Figure 2a–b. (b) PD distributions before (black) and after (red) learning. Note that none of the learning sessions altered directional representation, in general. (TIF) [file pone.0032986.s004.tif]
